# Low availability of carnitine precursors as a possible reason for the diminished plasma carnitine concentrations in pregnant women

**DOI:** 10.1186/1471-2393-10-17

**Published:** 2010-04-25

**Authors:** Robert Ringseis, Nicole Hanisch, Gregor Seliger, Klaus Eder

**Affiliations:** 1 Institute of Animal Nutrition and Nutrition Physiology, Justus-Liebig-University, Giessen, Germany; 2 Institute of Agricultural and Nutritional Sciences, Martin-Luther-University Halle-Wittenberg, Halle, Germany; 3 Department of Obstetrics and Gynecology, St. Elisabeth Hospital, Halle, Germany

## Abstract

**Background:**

It has been shown that plasma carnitine concentrations decrease markedly during gestation. A recent study performed with a low number of subjects suggested that this effect could be due to a low iron status which leads to an impairment of carnitine synthesis. The present study aimed to confirm this finding in a greater number of subjects. It was moreover intended to find out whether low carnitine concentrations during pregnancy could be due to a reduced availability of precursors of carnitine synthesis, namely trimethyllysine (TML) and γ-butyrobetaine (BB).

**Methods:**

Blood samples of 79 healthy pregnant women collected at delivery were used for this study.

**Results:**

There was only a weak, non-significant (P > 0.05), correlation between plasma concentration of ferritin and those of free and total carnitine. There was no correlation between other parameters of iron status (plasma iron concentration, hemoglobin, MCV, MCH) and plasma concentration of free and total carnitine. There were, however, significant (P < 0.05) positive correlations between concentrations of TML and BB and those of free and total carnitine in plasma.

**Conclusions:**

The results of this study suggest that an insufficient iron status is not the reason for low plasma carnitine concentrations observed in pregnant women. It is rather indicated that low plasma carnitine concentrations are caused by a low availability of precursors for carnitine synthesis during gestation.

## Background

Carnitine (L-3-hydroxy-4-N-N-N-trimethylaminobutyrate) is an essential metabolite, which has a number of indispensable functions in intermediary metabolism. The most prominent function lies in its role in the transport of activated long-chain fatty acids from the cytosol to the mitochondrial matrix where β-oxidation takes place. Other functions of carnitine include the transfer of products of peroxisomal β-oxidation to the mitochondria for oxidation in the citrate cycle, the modulation of the acyl-CoA/CoA-ratio and the storage of energy as acetylcarnitine [1-3]. All tissues that use fatty acids as a fuel source require carnitine for normal function. Carnitine is derived from dietary sources and endogenous biosynthesis [4,5]. Carnitine biosynthesis involves a complex series of reactions involving several tissues [6]. Lysine provides the carbon backbone of carnitine. Lysine in protein peptide linkages undergoes methylation of the ε-amino group to yield trimethyllysine (TML), which is released upon protein degradation. The released TML is further oxidised to γ-butyrobetaine (BB) by the action of trimethyllysine dioxygenase (TMLD), 3-hydroxy-N-trimethyllysine aldolase and 4-N-trimethylaminobutyraldehyde dehydrogenase (TMABA DH). BB is hydroxylated by γ-butyrobetaine dioxygenase (BBD) to form carnitine. In humans, this last reaction occurs primarily in liver and kidney [6]. Previous studies have shown that the availability of BB is limiting for carnitine synthesis while the activity of BBD is normally in excess of the amount of carnitine formed [7].

In non-pregnant, fertile women average plasma total carnitine concentration is around 40 μmol/L [8,9]. During gestation, plasma carnitine concentrations are strongly decreasing. At the time of delivery, plasma carnitine concentrations are decreased to about half of the concentrations of non-pregnant women [8-14], even though dietary carnitine intake increases as gestation proceeds [15]. Similar low carnitine concentrations are only found in patients with carnitine deficiency [16]. It is unclear whether such low carnitine concentrations in pregnant women have adverse effects on their metabolism or on the metabolism of the fetus. Nevertheless, due to the important functions of carnitine, it is possible that the low carnitine status during gestation could have beneficial effects on pregnant women and their fetuses.

The reasons for these low plasma carnitine concentrations are currently largely unknown. Recently, we investigated the effect of supplementation of carnitine during pregnancy on plasma carnitine concentrations [14]. In that study, we observed also a positive relationship between parameters of iron status (MCV, MCH, ferritin) and plasma carnitine concentration in the control group consisting of 13 pregnant women. This relationship seems plausible as two enzymes involved in carnitine synthesis, namely TMLD and BBD, contain iron with a catalytic function [17,18]. Hence, we suggested that low plasma carnitine concentrations are caused by a reduced carnitine synthesis due to a reduced activity of iron-dependent enzymes involved in carnitine synthesis. However, the low number of subjects considered in this study constitutes a clear limitation for the validity of the relationship between iron and carnitine in pregnant women.

In order to confirm a possible role of the iron status for plasma carnitine concentrations during gestation, we examined in the present study parameters of iron status and carnitine concentrations in plasma of 79 women at delivery. To find out whether a reduced availability of metabolic precursors could be limiting for carnitine synthesis, we determined also the concentrations of BB and TML in the plasma samples.

## Methods

### Subjects

Convenient blood samples from 79 pregnant women of an age between 20 and 43 years [25.8 ± 12.8 (SD) years], a height of 1.67 ± 0.07 (SD) m and a body mass index of 27.8 ± 4.0 (SD) kg/m^2 ^were included in this study. The plasma samples included those which were collected for routine blood analysis during hospitalization at the maternity units of St. Elisabeth and St. Barbara Hospital in Halle (Saale), Germany at delivery, between October and December 2008. No exclusion criteria such as gestational age, medical history, dietary habits or the intake of nutritional supplements were considered. The study was approved by the Research Ethics Committee of the hospital. Sample collection followed ethical principles for medical research according to the World Medical Association Declaration of Helsinki.

### Sample analysis

Plasma (20 μl) was added with methanol containing the internal standard. A 1100-er series HPLC (Agilent Technologies, Waldbronn, Germany) equipped with a Kromasil 100 column (5 μm particle size, 125 mm length, 2 mm internal diameter, CS-Chromatographie Service, Langerwehe, Germany) and an API 2000 LC-MS/MS-System (Applied Biosystems, Darmstadt, Germany) were used for quantification of free carnitine, acetyl carnitine, propionyl carnitine, BB and TML. For detection, the analytes were ionized by positive ion (5500 V) electrospray. As eluents, methanol and a methanol:water:acetonitrile mixture (50:45:5) were used (Hirche *et al*., 2007). Total carnnitine was caluculated as the sum of free, acetyl and propionyl carnitine. A full blood count (XE-2100; Sysmex GmbH, Norderstedt, Germany), including hemoglobin (Hb), MCV and MCH, was performed at the central labor of the hospital. Serum ferritin was determined with commercial enzyme linked immune sorbent assay kit (Alpha Diagnostic International, ELISA Kit Cat. No. 1210, San Antonio, Texas, USA). Concentration of plasma total iron was performed with a kit reagent [Quanti Chrom™ Iron Assay Kit (DIFE-250), Bio Assay Systems, Hayward, CA, USA].

### Statistical methods

Correlation analyses were performed using the Minitab Statistical Software (Release 13, Minitab Inc., State College, PA, USA). R-squared and adjusted R-squared were calculated with the linear regression tool using ferritin, TML or BB as predictors and free carnitine or total carnitine as responses. Correlations were considered significant for P < 0.05.

## Results

Concentrations of free carnitine, carnitine esters, total carnitine and the carnitine precursors TML and BB as well as parameters of iron status of the subjects are shown in Table 1. Concentrations of carnitine and the carnitine precursors as well as iron concentration showed a larger variation between the subjects than those of MCV, MCH, Hb and ferritin.

**Table 1:** Concentrations of carnitine, carnitine precursors and parameters of iron status in plasma of the subjects (n = 79)

	Mean ± SD	Minimum - Maximum
Free carnitine, plasma (μmol/l)	12.8 ± 2.91	7.67-22.6
Carnitine esters, plasma (μmol/l)	1.88 ± 0.64	0.92-3.64
Total carnitine, plasma (μmol/l)	14.7 ± 3.34	9.09-25.7
TML, plasma (μmol/l)	0.33 ± 0.12	0.18-0.89
BB, plasma (μmol/l)	0.33 ± 0.10	0.19-0.65
MCV (fl)	89.0 ± 5.09	74.6-100.6
MCH (fmol/l)	1.90 ± 0.13	1.55-2.17
Hb (mmol/l)	7.64 ± 0.77	5.0-9.9
Ferritin, plasma (μg/l)	0.47 ± 0.05	0.38-0.68
Fe, plasma (mg/l)	2.27 ± 1.12	1.03-4.63

**Figure 1:**
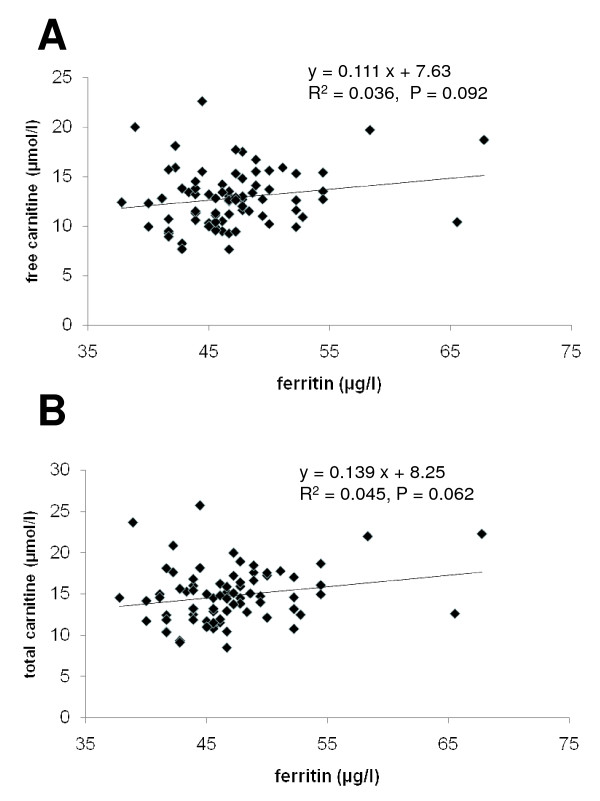
**Correlation between plasma ferritin concentration and plasma concentrations of free carnitine (A) and total carnitine (B)**.

There was a tendency for a positive relationship between plasma ferritin concentration and plasma concentrations of free carnitine (P = 0.09) and total carnitine (P = 0.06) (Figure 1). There were, however, no correlations between all the other parameters of iron status (plasma Fe concentration, MCV, MCH, Hb) and plasma concentrations of free and total carnitine (data not shown).

**Figure 2:**
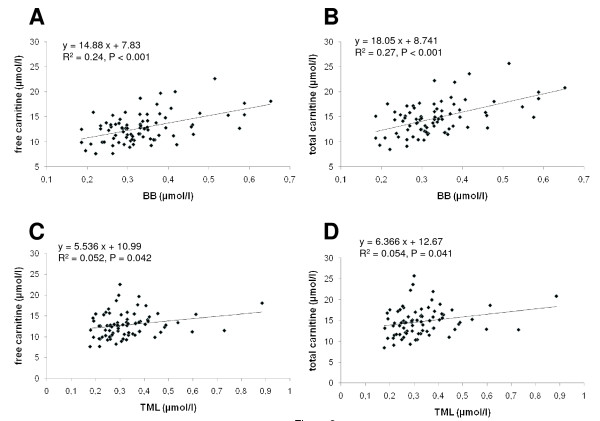
**Correlation between plasma concentrations of TML and plasma concentrations of free (A) and total carnitine (B), and between BB and plasma concentrations of free (C) and total carnitine (D)**.

There was a significant positive correlation between plasma concentration of TML and BB (P < 0.001; data not shown). Moreover, there were significant positive correlations between plasma concentrations of the carnitine precursors TML and BB and plasma concentrations of free and total carnitine (Figure 2). While the correlation between TML and carnitine concentration was relatively weak, although being statistically significant (P < 0.05), that between BB and carnitine concentration was much stronger. There were, however, no significant correlations between parameters of iron status and concentrations of TML and BB (data not shown).

## Discussion

A recent study with a lower number of subjects indicated that low plasma carnitine concentrations observed in pregnant women could be due to a low iron status [14]. One aim of the present study was to proof this indication in a greater number of pregnant women. Therefore, we considered plasma samples of 79 pregnant women at delivery. Average plasma total carnitine concentrations at delivery, being around 15 μmol/l, are in well agreement with those of our previous study [14] and several other studies [8-13,15]. Thus, the finding that pregnant women have a very low carnitine status at delivery is confirmed by the present study. Plasma carnitine concentrations below 20 μmol/L have been even considered as a marker of carnitine deficiency [19] although it is unknown whether these low carnitine concentrations have adverse effects on the metabolism of women or their fetuses.

In the present study, we could not support the previous finding that a low iron status is the main reason for the low plasma carnitine concentrations observed at delivery. The correlation between plasma ferritin and carnitine concentrations in this study was much weaker than that observed in the previous study [14], and moreover failed to be statistically significant. The lack of correlations between other parameters of iron status and plasma carnitine concentrations also indicates that an insufficient iron status can be ruled out as the main reason for low carnitine concentrations. Although unlikely, we cannot exclude the possibility that a significant correlation could not be demonstrated because of the inclusion of pregnant women with abnormal dietary habits, e.g. intake of nutritional supplements, physical activity preceding delivery, pathological abnormalities or different gestational ages. In this study, no subjects were excluded from this study based on gestational age, dietary habits, physical activity or medical history.

On the other hand, we observed for the first time significant correlations between concentrations of the metabolic carnitine precursors TML and BB and plasma carnitine concentrations. Previously, it has been shown that the availability of BB in liver and kidneys is limiting for carnitine biosynthesis in humans while the activity of BB in these tissues is normally in excess of the amount of carnitine formed [7]. This means that low plasma concentrations of carnitine in pregnant women could be the result of a diminished carnitine biosynthesis due to the lack of sufficient BB available as a precursor. This suggestion is supported by the fact that plasma BB concentrations in pregnant women are indeed lower than in non-pregnant subjects [14,20]. In our previous study, plasma BB concentrations in women at delivery (0.31 μmol/L) were similar to those observed in the present study while they were rising during the lactation period to a level of 0.54 μmol/L at day 28. The increase of plasma BB from delivery to day 28 of lactation was paralleled by an increase of plasma carnitine concentration [14].

BB is derived from TML which acts as the first precursor in the carnitine biosynthesis pathway. Accordingly, the finding of a positive relationship between TML and BB concentrations is plausible. This correlation indicates that the low concentration of BB, the direct precursor of carnitine, is due to a low availability of TML. TML is formed by methylation of lysine in protein peptide linkages and then released upon protein degradation. Indeed, plasma concentrations of TML like those of BB are reduced in pregnant women compared to non-pregnant subjects [14,20]. Diminished plasma TML concentrations in pregnant women may be the result of a reduced rate of protein breakdown since gestation is an anabolic state with an increase in maternal fat and protein stores [21]. Alternatively, the possibility that TML concentration was due to a reduced methylation of protein bound lysine cannot be ruled out.

## Conclusion

This study does not confirm that low carnitine concentrations observed in pregnant women are due to an insufficient iron status. However, a positive correlation between plasma concentration of BB and that of carnitine has been observed. This suggests that low plasma concentrations of carnitine in pregnant women may be the result of a diminished carnitine biosynthesis due to the lack of sufficient BB available as a precursor.

## Competing interests

The authors declare that they have no competing interests.

## Authors' contributions

NH carried out the analyses, performed the statistical analyses and participated in drafting the manuscript. GS provided advice on this concept and was responsible for the study coordination, proband care and the sample collection. RR provided advice on the study concept, supervised the analyses and participated in drafting the manuscript. KE designed the study concept, supervised the study and drafted the manuscript. All authors read and approved the final manuscript.

## Pre-publication history

The pre-publication history for this paper can be accessed here:

http://www.biomedcentral.com/1471-2393/10/17/prepub
